# Impact of Hemp Flour on the Nutritional, Sensory and Functional Characteristics of Wheat and Whole Wheat Muffins

**DOI:** 10.3390/foods14203578

**Published:** 2025-10-21

**Authors:** Andreea-Lavinia Mocanu, Alina Alexandra Dobre, Corina-Alexandra Stroe, Cătălina-Beatrice Poteraș, Elena-Loredana Ungureanu, Gabriel Mustatea, Gabriela Daniela Criveanu-Stamatie, Șerban Eugen Cucu, Sabina Andreea Bobea, Cristian Florea, Mihai-Bogdan Nicolcioiu, Raluca Stan

**Affiliations:** 1 Department of Organic Chemistry, Faculty of Chemical Engineering and Biotechnology, National University of Science and Technology Politehnica Bucharest, 1-7 Gh. Polizu Street, 011061 Bucharest, Romania; andreea.mocanu1@yahoo.com (A.-L.M.); corina10_2010@yahoo.com (C.-A.S.); beatrice.poteras@bioresurse.ro (C.-B.P.); cristian.florea@bioresurse.ro (C.F.); 2 National Research and Development Institute for Food Bioresources, 5 Ancuța Băneasa Street, District 2, 020323 Bucharest, Romania; alina.dobre@bioresurse.ro (A.A.D.); elena.ungureanu@bioresurse.ro (E.-L.U.); gabi.mustatea@bioresurse.ro (G.M.); gabriela.stamatie@bioresurse.ro (G.D.C.-S.); serban.cucu@bioresurse.ro (Ș.E.C.); sabina.bobea@bioresurse.ro (S.A.B.); bogdan.nicolcioiu@bioresurse.ro (M.-B.N.); 3 Faculty of Biotechnology, University of Agronomic Sciences and Veterinary Medicine of Bucharest, 011464 Bucharest, Romania

**Keywords:** hemp flour, muffins, wheat vs. whole-wheat, texture profile analysis, colorimetry, sensory evaluation, nutritional enrichment, functional bakery, fiber fortification

## Abstract

The growing consumer demand for plant-based, protein- and fiber-enriched foods has encouraged the incorporation of novel functional ingredients into bakery products. Hemp flour (HF), obtained from cold-pressed hemp seeds, represents a sustainable ingredient rich in proteins, dietary fibers, lipids, and bioactive compounds, making it suitable for nutritional fortification. This study investigated the impact of HF addition (5–40%) on the quality of muffins prepared with wheat flour (WF) and whole wheat flour (WWF). An initial hedonic sensory evaluation identified 5–20% HF as the most acceptable substitution range, which was then subjected to detailed physicochemical, sensory, textural, colorimetric, and microbiological analyses. Incorporation of HF significantly increased protein (up to +44%), fiber (up to +172%), and ash (up to +76%) contents, while decreasing moisture (−39%). Both WF and WWF muffins darkened with HF incorporation, with a greater lightness reduction in WF. Texture changes (increased firmness and gumminess) were more pronounced in WF muffins. Sensory analysis revealed that WF muffins were best accepted at 10–15% HF, whereas WWF muffins maintained good acceptability up to 20% HF, indicating better integration of HF in the whole grain matrix. All samples complied with microbiological safety requirements. Overall, the optimal substitution level was 10–15% HF in WF muffins and 20% HF in WWF muffins, demonstrating that HF can enhance the nutritional profile of muffins while maintaining acceptable technological and sensory properties in a matrix-dependent manner.

## 1. Introduction

Hemp (*Cannabis sativa* L.) is a versatile and historically significant herbaceous plant, originating from Central Asia [1]. For centuries, it has played an important role in many cultures, valued for its wide range of applications. Traditionally, hemp was recognized for its therapeutic properties and commonly used in folk medicine to alleviate various ailments [2]. It has also served as an important source of strong, durable fibers, historically used in the production of textiles, ropes, and other essential materials—making it a cornerstone of early industries. In addition to its use in textiles, hemp provides a rich supply of cellulosic and woody fibers utilized in paper manufacturing and as components in sustainable construction materials. What distinguishes hemp is its remarkable phytochemical composition, including cannabinoids, terpenes, and flavonoids—bioactive compounds with potential health-promoting effects [3,4,5,6,7]. These compounds contribute to the plant’s growing importance in modern sectors such as food, cosmetics, and pharmaceuticals [8,9]. Owing to its multifunctional applications and rich chemical profile hemp continues to attract scientific attention as one of the most valuable and sustainable crops in human history [10,11,12].

Owing to its diverse applications and complex chemical composition, hemp has attracted scientific interest for centuries. Historically, it played a vital role across multiple industries, and today it continues to be investigated for its health benefits and potential as a sustainable resource. Its exceptional versatility, combined with a low environmental footprint, has established hemp as one of the most valuable crops cultivated by humans. As global interest in renewable and eco-friendly materials increases, hemp remains at the forefront of agricultural and scientific research, highlighting its enduring relevance from the past to the present and into the future [1,3,7,11,13,14].

Hemp flour (HF) is a non-traditional, gluten-free, plant-based ingredient that has gained increasing attention in the food industry due to its high nutritional value and versatility. Its incorporation into bakery products has expanded in recent years, driven by the growing consumer demand for healthier and more sustainable food alternatives. HF is produced from hemp seeds, which are naturally rich in protein, dietary fiber, and essential fatty acids, making it a promising substitute for conventional cereal flours. A substantial portion of HF is derived from by-products of hemp oil extraction, such as hemp seed cake and hemp protein, which are further processed into flour—contributing to a circular and resource-efficient production model [15,16,17]. HF is a nutrient-dense, providing protein, dietary fibers, essential fatty acids, vitamins, and minerals [18]. Its nutritional composition and functional properties make it suitable for a wide range of food applications, including baked goods, snacks, and other processed products. As interest in sustainable and alternative food sources continues to grow, HF is increasingly recognized as a valuable ingredient for developing innovative, health-oriented products [15,18,19]. Its inclusion can enhance protein content, provide beneficial omega-3 and omega-6 fatty acids, and impart a characteristic nutty flavor and darker color to baked products [15,18,19,20].

Several studies have investigated the incorporation of hemp-derived ingredients into bakery products to enhance their nutritional and functional properties. The addition of HF has been reported in bread, cookies, and other baked goods, resulting in higher protein, fiber, and mineral content, as well as improved antioxidant potential [21,22,23,24]. Merlino et al. [25] examined the enrichment of gnocchi with hemp seed flour and observed significant improvements in the nutritional composition, particularly in protein and fiber levels, as accompanied by notable modifications in texture and sensory attributes. Similarly, Du et al. [26] investigated the fortification of muffins with hempseed protein isolate (HPI) and reported enhanced protein content and antioxidant activity, along with distinct changes in texture and sensory properties.

Beyond hemp-based ingredients, numerous studies have examined the fortification of muffins and other baked goods with alternative plant-based flours such as chickpea, lentil, quinoa, soy, flaxseed, and chia. These ingredients have been shown to enhance the protein, fiber, and mineral contents of bakery products while also affecting their color, texture, and sensory attributes [27,28,29,30]. Collectively, these studies emphasize the versatility and potential of plant-derived ingredients in formulating functional bakery products with improved nutritional and technological characteristics.

However, the study by Du et al. [26] differs fundamentally from the present work, as HPI represents a purified protein fraction, whereas HF is a complex by-product obtained from the cold pressing of hemp seeds for oil extraction. HF contains not only proteins but also dietary fiber, lipids, and other bioactive compounds. This broader composition influence both the technological performance and sensory characteristics of the final product, underscoring the importance of investigating the direct application of whole hemp flour in baked goods.

The most distinctive feature of this research is the direct comparison between two commonly used base flours: WF and WWF. These flours differ substantially in composition and technological behavior, with WWF containing higher levels of fiber, minerals, and bioactive compounds, whereas WF contributes to a lighter texture and milder flavor. Such compositional differences strongly influence the interaction of HF within each matrix, resulting in variations in dough properties, sensory attributes, and overall product quality. By incorporating HF into both WF- and WWF-based muffin formulations, this study offers a comprehensive perspective on how the type of base flour affects the functionality and consumer acceptability of the final product.

Muffins were prepared by substituting WF and WWF with HF at levels ranging from 0% to 40%. The samples were evaluated through hedonic and descriptive sensory tests, Texture Profile Analysis (TPA), CIE Lab* color measurements, physicochemical characterization, and microbiological assessment. Detailed analyses focused on the 5% to 20% HF range, as identified by the preliminary hedonic screening. The objective of this study was to quantify matrix-specific trade-offs between quality and nutritional enhancement and to determine the optimal HF substitution levels for WF- and WWF-based muffins.

## 2. Materials and Methods

### 2.1. Preparation of Samples

The muffin samples were prepared using two base formulations: one with refined WF and another with WWF. In both cases, HF partially replaced the base flour at substitution levels of 5%, 10%, 15%, 20%, 30% and 40%. Control samples contained only the corresponding base flour, while the experimental samples incorporated the specified HF percentages.

Commercial WF type 650 (ash 0.65%, moisture ≤ 14%) and WWF (ash 1.80%, moisture ≤ 14%) were obtained from Boromir S.A. (Buzău, Romania), batches WF—0423 and WF—0423, respectively. HF was sourced from Canah Internationals, Romania, with the following proximate composition per 100 g: fat—8.2 g (of which saturated—1.5 g, monosaturated—2.6 g, polysaturated—4.0 g), carbohydrates—3.8 g (of which sugars—3.5 g), fiber—48 g, protein—34 g, and salt 0.03 g. All other ingredients (sugar, eggs, butter, baking powder, and flavor) were purchased from local suppliers.

**Figure 1:**
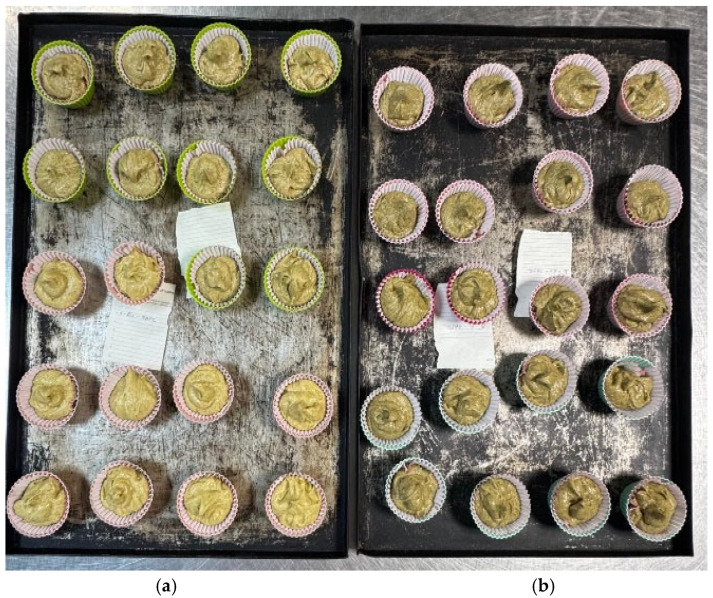
Preparation stage of muffin samples supplemented with different concentrations of hemp flour (HF). Each tray corresponds to a different base flour formulation: (**a**)—WF, and (**b**)—WWF.

The formulations consisted of flour (WF or WWF), sugar, eggs, butter (82% fat), baking powder, and flavor. The preparation process began with creaming the butter and sugar until a homogeneous mixture was obtained. Eggs were added one at a time, followed by flavor. The pre-mixed dry ingredients (flour and baking powder) were gradually added to the wet mixture, and the batter was mixed until uniform. Batter portions of 48 g were distributed evenly into paper molds to ensure uniformity across all formulations. The portioning and pre-backing stage of the muffin samples is illustrated in Figure 1.

The batter corresponding to each formulation (as presented in Table 1) was baked in silicon trays at 190 °C for 35 min.

After baking, the muffins were allowed to cool at room temperature and then stored under controlled conditions until analyses. Each sample was individually packed in sealed plastic bags to prevent moisture loss and contamination. All experimental products were Prepared and analyzed in duplicate.

### 2.2. Analyses Performed

Sensory analysis was conducted to evaluate both the overall acceptability and the detailed sensory characteristics of muffins prepared with WF and WWF enriched with different levels of HF. Two complementary tests were performed: a hedonic test to assess consumer preference and overall liking, and a descriptive sensory analysis using a trained panel to quantify specific sensory attributes.

All sensory evaluations were carried out in a controlled sensory laboratory in accordance with the General Data Protection Regulation (EU 2016/679) and ethical research standards. Participants provided informed consent before testing, and all collected data were anonymized.

#### 2.2.1. Preferential (Hedonic) Sensory Analysis

The hedonic analysis was conducted to assess the total acceptability of muffins formulated with WF and WWF, incorporating different levels of HF (5%, 10%, 15%, 20%, 30%, and 40%).

A group of 34 untrained consumers participated in the study, representing potential target consumers for hemp-enriched muffins. The evaluation was conducted under controlled conditions to minimize external influences on perception. Each participant was asked to assess overall acceptability using a 9-point hedonic scale, where 9 = Like extremely, 5 = Neither like nor dislike and 1 = Dislike extremely [30,31,32,33,34,35]. Participants were not given additional details about the formulation to prevent bias or preconceptions from influencing the results.

The muffins were prepared using standardized baking conditions, ensuring consistency across all formulations. Samples were coded with randomized three-digit numbers and were presented in a random order to prevent positional effects or preference biases: the samples were served at room temperature on neutral white plates to avoid visual bias from dishware color; the portion size was uniform to ensure an equal evaluation of texture, taste, and mouthfeel and participants were instructed to rinse their palate with water between samples to prevent taste carryover effects.

#### 2.2.2. Descriptive Sensory Analysis

This analysis focused on quantifying specific sensory attributes of the muffins using a trained panel of assessors.

Muffins were prepared under standardized conditions and served as uniform portions under neutral lighting. Samples were blind-coded with randomized three-digit numbers and presented in random order. Panelists rinsed their mouths with water between samples to prevent carryover effects.

Each sample was evaluated for key sensory attributes, including appearance (crust and crumb color, pore structure), texture (elasticity, firmness, cohesiveness, moisture), flavor and taste (baked aroma, hemp intensity, sweetness, bitterness, astringency, lubricity), and aftertaste. Attributes were rated on an intensity scale, where higher values indicated stronger perception [34,36].

#### 2.2.3. Texture Analysis

The texture analysis was performed to evaluate the mechanical properties of samples obtained. The goal of this analysis was to quantify textural changes induced by HF addition using instrumental texture profile analysis (TPA), providing objective data on the muffins’ firmness, cohesiveness, elasticity, and gumminess.

Texture analysis was performed using an INSTRON 5944 Texture Analyzer (Instron, Norwood, MA, USA) equipped with a 50 N load cell. Cylindrical muffin samples were subjected to a double compression test to 50% deformation at a crosshead speed of 1 mm s^−1^, simulating the first and second bite. Firmness, cohesiveness, springiness, and gumminess were determined automatically using the Bluehill software (V4.55). All measurements were conducted at 20 ± 1 °C in triplicate, and results were expressed as mean ± SD. The procedure followed standard TPA methodology described by Refs. [37,38,39].

#### 2.2.4. Color Analysis

The color analysis was conducted to assess the impact of HF incorporation (5–20%) on the visual appearance of muffins formulated with WF and WWF. The objective was to quantify color differences caused by varying HF concentrations using the CIE Lab color system, which provides an objective measure of lightness (L), redness (a*), and yellowness (b*).

Color parameters (L*, a*, b*) were measured on the muffin crumb using a Minolta CM-5 colorimeter (Konica Minolta, Tokyo, Japan) operating in the CIE Lab system (illuminant D65, 10° observer angle, reflectance mode). Three measurements were taken at random points on each sample, in triplicate, and mean ± SD values were reported. Total color difference (ΔE*) was calculated according to Ref. [40], following the procedure described by Refs. [41,42].

#### 2.2.5. Microbiological Analysis

Microbiological analysis was performed to assess the safety and hygiene of muffins containing different levels of hemp flour (HF: 5–20%) prepared with WF and WWF. The presence of yeasts, molds, and Enterobacteriaceae was determined according to ISO standards for bakery products.

Yeasts and molds were enumerated using the spread-plate method on DG-18 agar, incubated at 25 °C for 5–7 days (ISO 21527-2:2008) [43], while Enterobacteriaceae were quantified on Violet Red Bile Glucose Agar (VRBGA) at 37 °C for 24 ± 2 h (ISO 21528-2:2004) [44]. All analyses were performed in triplicate, and results were expressed as colony-forming units per gram (CFU/g). Counts below 10 CFU/g were considered below the detection limit.

#### 2.2.6. Physicochemical Analyses

All reagents utilized in the analyses were procured from Merck and conformed to the respective analytical standards. Merck’s analytical reagents are specified according to the American Chemical Society (ACS) standards and the European Pharmacopoeia (Reag. Ph Eur.), ensuring high purity and suitability for precise analytical applications.

Moisture, ash, crude fiber, protein, total sugars, and lipid contents were determined according to ISO and SR standards applicable to bakery and cereal-based products [ISO 712:2009 [45,46] (the additional reference supports a similar procedure applied in enriched bakery prod-ucts); ISO 2171:2007 [47]; SR EN ISO 6865:2002 [48]; SR EN ISO 20483:2014 [49]; SR 91:2007 [50,51] (the latter reference describes comparable analytical conditions for functional bakery for-mulations); ISO 11085:2015 [52]]. Moisture content was measured by thermogravimetric drying at 130 °C [45,46]; ash by incineration at 900 °C [47]; crude fiber via acid-alkaline digestion and gravimetric quantification [48]; protein using the Kjeldahl method (N × 6.25 conversion factor) [49]; total sugars by Fehling-Lane-Eynon titration [50,51]; and fat content by Soxhlet extraction with petroleum ether [52]. All analyses were performed in duplicate, and results were expressed on a dry weight basis.

### 2.3. Statistical Analysis

Data were analyzed using Minitab 21 and XLSTAT (version 2023). Normality (Shapiro–Wilk) and homoscedasticity (Levene) were verified before applying one-way or two-way ANOVA, followed by Tukey’s HSD test (α = 0.05) where applicable. Results are expressed as mean ± SD, with superscript letters (a, b) indicating homogeneous groups. Hedonic data were processed using a General Linear Model (GLM) with Sample and Choice as fixed factors and expressed as preference percentages. PCA and AHC were applied for texture and color datasets to identify multivariate relationships, while microbiological results were evaluated descriptively against bakery safety standards. The corresponding PCA and AHC plots are provided in the Supplementary Materials (Figures S1–S16).

## 3. Results and Discussion

This study investigates the impact of HF on muffin quality through a comprehensive set of analyses. First, the preparation of samples is detailed, followed by a sensory evaluation, including hedonic analysis for overall acceptability and descriptive sensory analysis. The physical properties of the samples are assessed through texture and color measurements, microbiological analysis to examine the presence of yeast, molds, and *Enterobacteriaceae*, physicochemical analyses to determine key compositional parameters, including moisture, ash, fiber, protein, fat, and sugar content.

### 3.1. Sensory Analyses

#### 3.1.1. Preferential (Hedonic) Sensory Analysis

**Figure 2:**
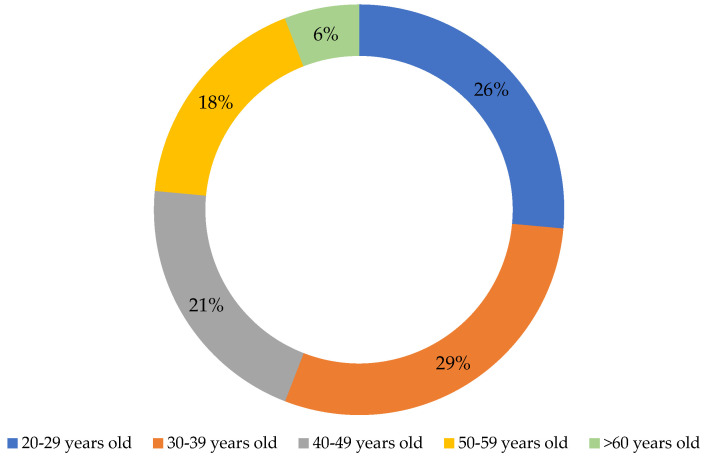
Age distribution of consumers participating in preferential sensory analysis of muffin samples prepared with wheat flour and whole wheat flour supplemented with different concentrations of hemp flour.

Hedonic screening was conducted with 34 consumers (73.5% women, aged 20–60 years—Figure 2.) to assess the acceptability of muffins containing 0–40% hemp flour (HF). Samples were randomly coded and evaluated under controlled conditions to avoid bias.

In order to determine the acceptability of the samples according to the concentration of hemp added, the consumers choose at the end for each group (samples with WF and samples with WWF, from the 6 samples only 4, in ascending order—from the one they liked the most to the one they liked the least). The results obtained are presented below, the exact number of selections and estimated preference percentages are also provided in Table 2.

**Figure 3:**
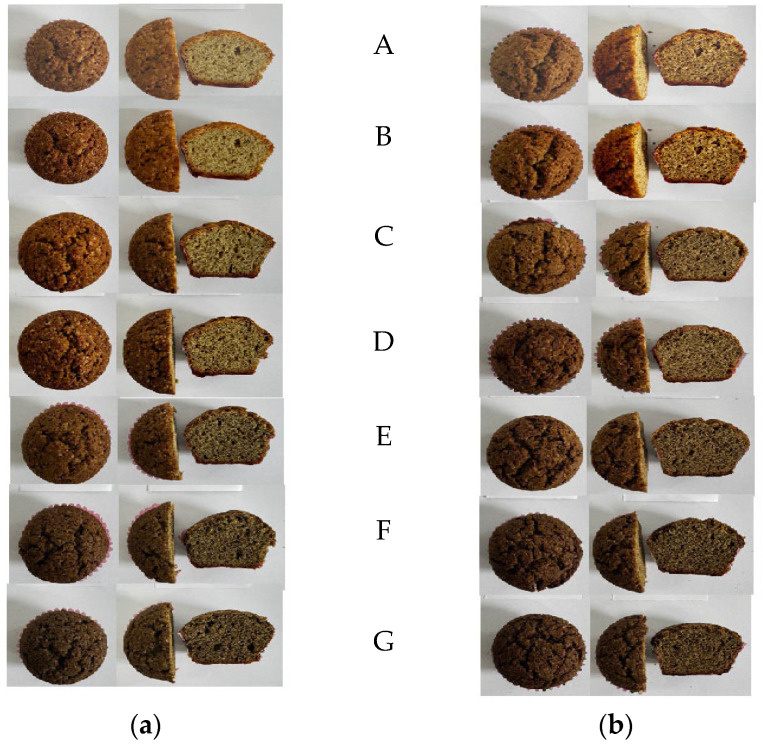
Visual appearance of muffins prepared with increasing hemp flour substitution levels. (**a**) wheat flour–based muffins, and (**b**) whole wheat flour–based muffins, each arranged by hemp flour content: (**A**) Control, (**B**) 5% hemp flour, (**C**) 10% hemp flour, (**D**) 15% hemp flour, (**E**) 20% hemp flour, (**F**) 30% hemp flour, and (**G**) 40% hemp flour.

Based on the results of the sensory evaluation conducted with participants, the most appreciated levels of HF addition were 5%, 10%, 15%, 20%, for both WF and WWF muffin formulations. Representative images of the muffins with different HF substitution levels are shown in Figure 3. Progressive darkening and structural differences are visible as HF concentration increases, corresponding to the sensory evaluation trends. These specific samples received the highest overall percentages—ranging from 73.5% to 82.4%—demonstrating optimal consumers acceptability.

The incorporation of hemp flour (HF) significantly influenced sensory perception, with concentrations between 5% and 20% yielding the most favorable results. These levels enhanced texture, flavor balance, and color without compromising overall acceptability, while higher additions (30–40%) were less appreciated due to denser texture, darker color, and stronger hemp flavor. Overall, muffins with whole wheat flour (WWF) achieved greater acceptability at higher HF concentrations than those with refined wheat flour (WF), indicating better integration of HF into the whole grain matrix. The findings highlight HF’s potential as a functional ingredient in bakery products and emphasize the importance of optimizing substitution levels to maintain desirable sensory properties and consumer satisfaction.

For both WF and WWF muffin formulations, HF enrichment up to 40% did not significantly affect consumer hedonic evaluations (WF: *p* = 0.267; WWF: *p* = 0.639). Statistical analysis was performed using the General Linear Model (GLM) in Minitab 19, with Sample and Choice as fixed factors. The hedonic test was based on consumer preference ranking, where panelists indicated their first to fourth choices among the samples.

Because the dataset represented ranking frequencies rather than continuous measurements, results were expressed as estimated preference percentages, without standard deviations or post hoc significance letters. These results indicate that hemp flour can be incorporated into muffin formulations at various substitution levels without negatively influencing overall consumer acceptability. Although no statistically significant differences were observed, descriptive trends in the mean preference percentages reveal subtle consumer tendencies that may guide future product optimization.

#### 3.1.2. Descriptive Sensory Analysis

**Figure 4:**
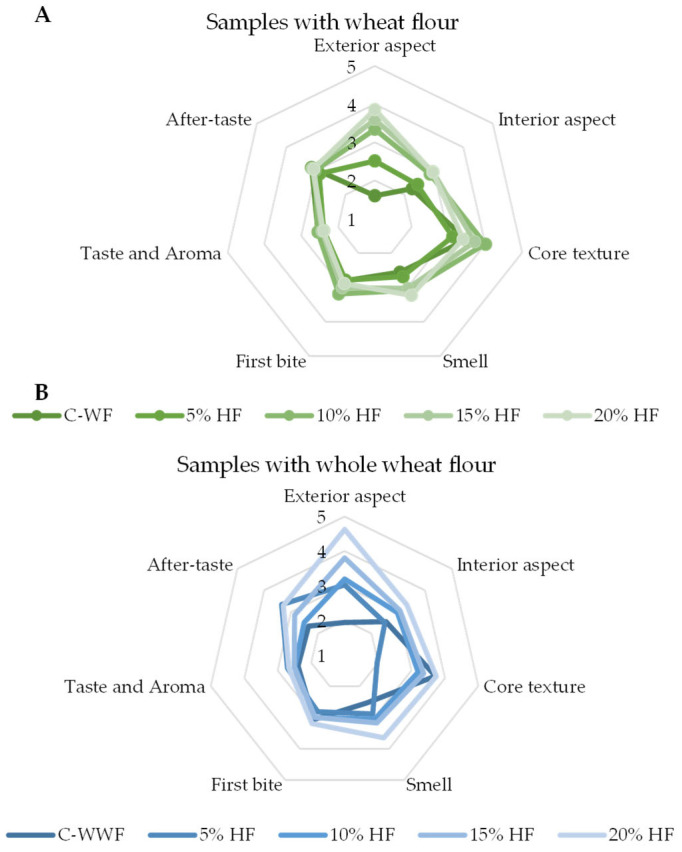
Descriptive sensory profile of muffin samples prepared with wheat flour and whole wheat flour supplemented with different concentrations of hemp flour. Panel A represents samples with wheat flour, and panel B represents samples with whole wheat flour. Statistical significance among formulations is provided in Table 3 (Tukey HSD, *p* < 0.05).

The sensory profiles of the selected WF and WWF muffin samples with different levels of HF are illustrated in figure below (Figure 4), based on the descriptive evaluation conducted by the trained panel.

To provide a clear understanding of the sensory differences across samples, the exact values used to construct the charts are detailed in the table below (Table 3).

The radar charts presented in Figure 4 illustrate the sensory profiles of the muffin samples with different concentrations of HF, based on a trained panel’s evaluation. Each axis represents a distinct sensory attribute, and the data points reflect the intensity scores assigned by the consumers. The right chart (blue) corresponds to the formulation set of samples obtained with WF, while the left chart (green) represents the samples obtained with WWF.

The comparative sensory evaluation of WF–HF and WWF–HF muffins revealed distinct patterns across attributes. WF samples exhibited greater variability in appearance, aroma, and taste, suggesting a stronger sensitivity of the refined wheat matrix to HF incorporation. In contrast, WWF formulations showed more consistent scores across all attributes, particularly for aroma and overall acceptability, indicating better structural and flavor integration of HF within the whole grain matrix. Moderate HF levels (5–20%) were the most appreciated in both matrices, while higher additions (30–40%) decreased texture softness and flavor balance. Overall, these results demonstrate that WWF supports higher HF incorporation with minimal sensory compromise, whereas WF requires more precise optimization to maintain product quality and consumer appeal.

The principal component analysis (PCA) explained 81.46% of the total variance across the first two components (F1 = 51.64% and F2 = 29.82%). Core texture and first bite were the most influential attributes along F2, while exterior aspect, interior aspect, and appearance aligned closely along F1. Samples enriched with 10–20% HF (both WF and WWF) grouped near the origin, indicating a balanced sensory profile. In contrast, the 5% WWF sample was positioned furthest from the cluster, suggesting notable sensory deviation.

The agglomerative hierarchical clustering (AHC) further confirmed these groupings, forming two major clusters: one with the higher HF samples (10–20%) and another with control and 5% WF samples, suggesting distinct sensory differentiation. The 5% WWF sample again appeared as an outlier.

The sensory evaluation revealed that HF substitution significantly influenced the sensory quality of muffins, depending on the type of base flour. Data obtained were analyzed by one-way ANOVA, followed by Tukey’s HSD post hoc test (α = 0.05), and results are presented as mean ± SD with superscript letters indicating homogeneous groups.

For WF muffins, no significant differences were observed among the substitution levels (*p* = 0.082), although samples containing 10–20% HF showed slightly higher mean scores across most sensory attributes. In contrast, WWF muffins exhibited significant differences (*p* = 0.015), with the 20% HF formulation receiving the highest sensory ratings and being significantly preferred over the control and 5% HF samples.

These results indicate that HF incorporation has a stronger positive effect on WWF formulations, with 20% HF representing the optimal enrichment level for overall sensory acceptability.

Sensory analysis confirmed that HF incorporation up to 15% maintained high acceptability, while higher levels reduced scores due to increased firmness, darker color, and stronger hemp flavor. These trends align with previous reports showing that moderate HF enrichment improves product quality, whereas excessive substitution negatively affects texture and taste [15,23]. Notably, the 20% HF WWF muffins achieved the highest sensory ratings, confirming that whole wheat flour integrates hemp flour more effectively than refined wheat, consistent with prior findings on matrix-dependent sensory behavior [53].

Overall, sensory evaluation demonstrated that the optimal HF substitution level depended on the flour matrix. WF muffins achieved their highest sensory scores at 10–15% HF, whereas WWF muffins maintained good acceptability up to 20% HF, with the 20% WWF sample significantly outperforming lower substitution levels. This suggests that the fiber- and pigment-rich whole wheat matrix allows better integration of hemp flour, yielding more stable texture, color, and flavor attributes compared to WF formulations.

### 3.2. Physical Properties

#### 3.2.1. Texture Measurements

The purpose of this study was to evaluate the impact of HF incorporation (5–20%) on the textural properties of muffins made with WF (Type 650) and WWF. The texture parameters analyzed included firmness, elasticity, cohesiveness, and gumminess, as these attributes play a crucial role in consumer acceptability and product quality.

A comparative analysis was conducted between muffins formulated with WF and those made with WWF, to assess how the differences in flour composition interact with increasing HF concentrations. The study aimed to determine the optimal HF percentage that maintains desirable textural properties while improving the nutritional profile of the final product.

The following section presents the detailed results of texture profile analysis (TPA), highlighting the key trends observed across different formulations and providing insights into how HF affects the structure, chewability, and mechanical properties of muffins.

The results of the TPA for muffins with WF and WWF, incorporating 5% to 20% HF, are summarized in Table 4. The data includes key texture parameters such as firmness (indicates the resistance of the muffins to deformation, representing their hardness), elasticity (refers to the capacity of the muffins to recover their shape after being compressed), cohesiveness (measures the ability of the muffin structure to remain intact after compression), and gumminess (represents the energy required to chew the muffins until they are ready for swallowing), allowing for a direct comparison between formulations. These values highlight the structural differences caused by flour type and HF concentration, providing insights into the textural changes and their potential impact on product quality.

The TPA results demonstrate the significant impact of HF substitution (5–20%) on the textural attributes of muffins, particularly in terms of firmness, cohesiveness, elasticity, and gumminess. These parameters are essential in determining the sensory perception and structural integrity of the final product, with variations reflecting changes in the underlying composition and interactions between proteins, fibers and starches.

Firmness decreased by 35% (WF) and 22% (WWF) at 5% HF, indicating that small amounts of hemp flour soften the muffin matrix—likely due to reduced gluten strength and starch dilution. At 10% HF, firmness increased by 91% (WF) and 92% (WWF), suggesting the formation of a denser structure as fibers interact with starch and limit expansion. This reinforcing effect weakened at 15% HF, where firmness dropped again by 35% (WF) and 13% (WWF), potentially due to excess fiber disrupting the matrix. At 20% HF, firmness stabilized in WF but increased by 17% (WWF), reflecting inconsistent structural behavior at higher fiber levels.

Cohesiveness dropped by 40% (WF) at 5% HF, showing that initial fiber addition weakens internal binding, possibly due to gluten dilution. However, WWF showed a slight increase, indicating some compensatory fiber interactions in the whole wheat matrix. At 10% HF, cohesiveness improved by 50% (WF) but declined by 36% (WWF), suggesting an unstable balance between gluten weakening and fiber-starch interaction. At 15% and 20%, cohesiveness declined further in both cases, implying that higher fiber content disrupts the internal network and increases fragility.

Elasticity remained stable in WF but decreased by 54% (WWF) at 5% HF, showing that fiber has a greater impact on the springiness of whole wheat muffins. At 10% HF, elasticity slightly decreased in WF but increased by 20% (WWF), pointing to mixed effects of fiber on structure recovery. At 15%, elasticity dropped by 23% (WF) but rose modestly by 5% (WWF), and at 20%, it increased significantly by 102% (WF) while remaining stable in WWF. These shifts suggest that higher HF levels can sometimes stiffen the structure, improving spring-back in some cases, but reducing flexibility overall.

Gumminess decreased by 60% (WF) and increased slightly in WWF at 5% HF, indicating that muffins became softer and easier to break down. At 10% HF, gumminess increased by 132% (WF) and 52% (WWF), reflecting the formation of a more compact, chewier texture. At 15%, it dropped by 71% (WF) while staying relatively stable in WWF, and at 20%, gumminess showed high variability, decreasing by 206% (WF) and increasing by 11% (WWF). These results suggest that excessive HF causes inconsistent texture due to uneven fiber distribution and a weakened gluten network.

For texture measurements, no univariate statistical comparison was performed, as the Texture Profile Analysis (TPA) generated multiple correlated parameters (firmness, cohesiveness, springiness, gumminess, etc.). To better understand the relationships among these attributes and their combined influence on sample structure, PCA and AHC were applied. These multivariate approaches provided an integrated overview of the textural behavior across formulations with varying hemp flour levels.

The PCA of textural parameters explained 80.82% of the total variance across the first two components (F1 = 53.06%, F2 = 27.77%). Firmness, cohesiveness, and gumminess were strongly associated with F1, while elasticity correlated mainly with F2. The PCA score plot shows that samples with higher HF additions were separated from controls and lower HF samples, particularly along F2 due to increased elasticity.

The AHC grouped the samples into two main clusters. One cluster included most of the WWF and higher HF samples, suggesting similar textural behavior, while the second cluster grouped the WF control and 20% HF samples, indicating closer textural similarity. Notably, 15% HF from WF group appeared as the most distinct sample, likely due to elevated elasticity and altered firmness.

Texture profile analysis showed that HF addition significantly increased firmness and gumminess while reducing elasticity, particularly at higher substitution levels. These changes can be attributed to the high fiber content of HF, which disrupts the gluten network and alters the structural matrix of the muffins. Ref. [24] reported comparable results in breads with 10–30% HF, observing increased hardness and darker crumb color. Similarly, [50] found that HF incorporation reduced elasticity and increased firmness, highlighting the weakening effect of fiber-rich ingredients on gluten development. Our findings confirm these reports and demonstrate that WWF muffins experienced slightly less texture deterioration than WF muffins, likely due to their naturally higher fiber and whole grain structure.

The effect of HF incorporation differed between WF and WWF muffins. In texture measurements, WF samples showed a stronger increase in firmness and gumminess with higher HF levels, reflecting greater gluten dilution and moisture loss. In contrast, WWF muffins exhibited smaller textural variations due to the buffering effect of bran fibers, which help retain water and stabilize structure (Table 4.).

#### 3.2.2. Color Measurements

Color is a key quality attribute in bakery products, influencing consumer perception and acceptability. The addition of HF to muffins is expected to induce color changes, depending on the concentration used and the interaction with WF or WWF. These changes occur due to the natural pigments present in HF, such as chlorophyll, polyphenols, and Maillard reaction products, which contribute to variations in lightness (L)—represents the brightness of the sample, with higher values indicating lighter muffins, redness (a*)—red-green axis determines the intensity of red or green hues, with positive values indicating redness and negative values indicating greenness, and yellowness (b*)—yellow-blue axis reflects the balance between yellow and blue tones, with positive values indicating more yellow shades.

The following section presents the color analysis results, highlighting the impact of HF concentration on the visual characteristics of muffins. This evaluation is essential in understanding consumer preferences and optimizing formulations to maintain desirable color properties while incorporating nutritional benefits from HF.

For WF muffins, lightness (L*) decreased from 72.54 (control) to 46.49 (20% HF), indicating a progressive darkening due to hemp pigments, polyphenols, and intensified Maillard reactions; redness (a*) dropped from 5.76 (control) to 3.34 (15% HF), then slightly increased to 3.57 (20%), suggesting a shift toward more neutral tones; yellowness (b*) declined consistently from 37.48 (control) to 23.90 (20% HF), showing a loss of yellow hue as HF concentration increased.

For WWF muffins, L* decreased from 59.93 (control) to 43.40 (20% HF), confirming the darkening trend; redness (a*) dropped from 6.73 to 4.68, with the most notable decline between 10% and 20% HF; yellowness (b*) initially rose at 5% HF (24.20), then decreased to 20.16 (20% HF), indicating that small HF additions can enhance yellow tones, but higher levels reduce color vibrancy.

The standard deviation for all samples analyzed, values for L, a*, and b* are generally low, indicating that the color measurements are consistent and reproducible. For samples with WF higher variability in a* and b* at 5% and 10% HF suggests that minor inconsistencies in pigment distribution may occur at lower concentrations. For samples with WWF at 20% HF, L and b* have higher standard deviations, suggesting greater color variation at high HF levels.

The following figure illustrates the impact of increasing HF levels on the color attributes of both formulations, highlighting the progressive darkening (lower L values), the reduction in red tones (a*), and the variation in yellow hues (b*). These findings are crucial in understanding the visual appeal and marketability of hemp-enriched bakery products.

Lightness (L*) decreased progressively in both WF and WWF muffins as hemp flour (HF) concentration increased, confirming a clear darkening effect. WF muffins showed a more pronounced change in brightness compared to WWF muffins, indicating that HF has a stronger visual impact on white flour-based formulations. Whole wheat muffins started darker and remained darker across all HF levels due to their naturally lower lightness.

Redness (a*) declined with increasing HF in both formulations, indicating a shift toward more neutral brown tones. The reduction was sharper in WF muffins, where redness dropped quickly and then stabilized. In contrast, WWF muffins showed a gradual and continuous decrease, retaining slightly more red tones throughout.

Yellowness (b*) also decreased with higher HF levels, indicating a fading of yellow hues. In WF muffins, the decline was consistent, while in WWF muffins, a slight initial increase was observed at low HF concentrations before the values declined. This suggests that low HF levels may briefly enhance yellow tones in whole wheat formulation before higher concentrations dull the color.

Overall, increasing HF led to darker, less vibrant muffins in both formulations, with more noticeable shifts in the WF samples. These color changes are likely influenced by hemp pigments, polyphenols, and Maillard reactions, and may affect consumer perception depending on expected visual cues for fiber-rich or whole grain products.

For color analysis, no statistical test (ANOVA or Tukey) was applied because the color parameters (L*, a*, b*) are interdependent and collectively describe a single perceptual attribute—overall color. Instead, Principal Component Analysis (PCA) and Agglomerative Hierarchical Clustering (AHC) were used to evaluate sample groupings and variation trends based on all color coordinates simultaneously. This multivariate approach provided a more comprehensive interpretation of the color changes induced by hemp flour addition.

The PCA of color parameters (L*, a*, b*) explained 96.05% of the total variance, with F1 (62.58%) primarily driven by the lightness (L*) and yellowness (b*) components, while F2 (33.47%) was influenced by redness (a*). The score plot showed that WF samples (especially 5%, 10% and 15%) were clearly separated along F1, indicating greater variation in lightness and yellowness, while WWF samples clustered closer to the center, suggesting more uniform color attributes.

The dendrogram generated by AHC revealed two major clusters. One included both control samples, showing their distinct color profiles compared to HF-enriched variants. The other cluster grouped HF-containing samples, with WF and WWF samples from 10% to 20% HF exhibiting similar chromatic behavior.

HF also caused darkening of the crumb color, attributable to its natural pigments, which aligns with observations by [24] and [15] in breads and gluten-free cakes, respectively. Texture profile analysis (TPA) revealed increased firmness and reduced elasticity at higher substitution levels, consistent with reports by [50], who noted weakening of the gluten network in HF-enriched breads. Our findings confirm that the structural changes induced by HF directly affect texture and visual quality, requiring careful optimization for consumer acceptance.

The compositional complexity of hemp flour explains many of the observed technological and sensory effects. The increased firmness and gumminess observed at higher HF levels (particularly above 15%) are associated with the high dietary fiber content, which enhances water-binding capacity but simultaneously dilutes the gluten network, reducing gas retention and leading to denser textures. These effects are consistent with the lower moisture levels measured in both WF and WWF formulations. Furthermore, the marked color changes (decreased L* and increased a*/b* values) reflect the contribution of natural pigments and polyphenolic compounds inherent to hemp flour, which impart a darker crumb and crust. In addition, the lipid and bioactive components of HF likely influenced aroma and flavor perception, as indicated by the descriptive sensory results showing more pronounced earthy and roasted notes at higher substitution levels. Collectively, these relationships demonstrate that the fiber, pigment, and bioactive fractions of HF, rather than protein enrichment alone, drive the distinct physicochemical and sensory trends observed—underscoring the novelty of whole-flour utilization compared with purified hemp protein isolates.

**Figure 5:**
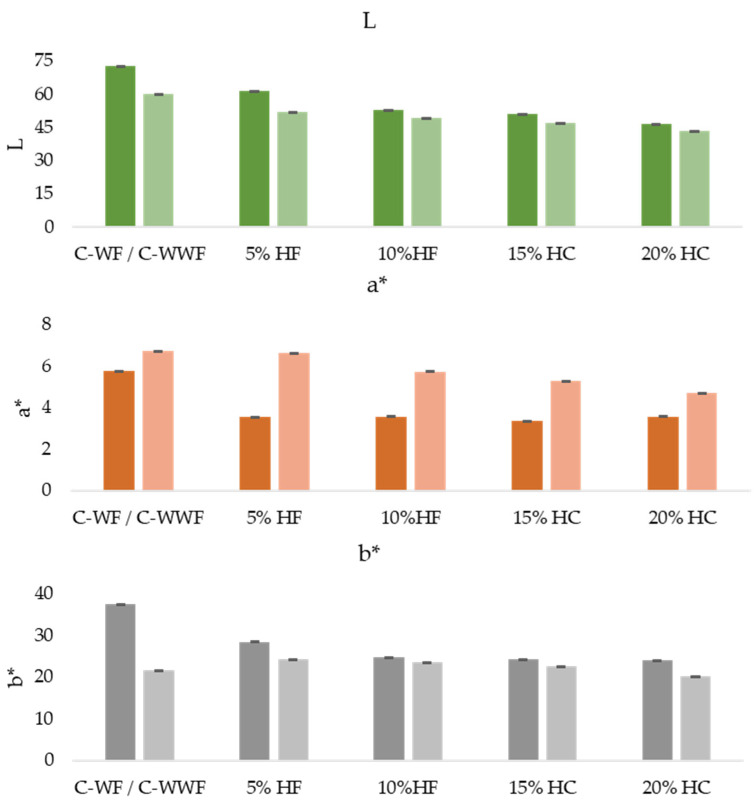
Effect of hemp flour addition on the color parameters (L, a, b*) of muffin samples prepared with wheat flour and whole wheat flour.

Color analysis revealed that both muffin types darkened with increasing HF concentration; however, the reduction in lightness (L*) was more pronounced in WF samples, while WWF muffins, initially darker, displayed smaller visual changes (Figure 5.). These trends highlight the combined effects of hemp pigments, polyphenols, and Maillard reactions, which influence appearance more strongly in lighter, refined matrices.

### 3.3. Microbiology Analysis

Microbiological evaluation was conducted to verify the hygienic quality and safety of the muffins formulated with wheat flour (WF) and whole wheat flour (WWF) containing different levels of hemp flour (HF; 5–20%). The analysis focused on the detection of yeasts, molds, and *Enterobacteriaceae*, key indicators of microbiological stability and product hygiene, in accordance with Regulation (EC) No. 2073/2005 and ISO standards [43,44].

All samples—both control and HF-enriched—showed counts below the detection limit (<10 CFU/g) for yeasts, molds, and *Enterobacteriaceae* (Table 5). These results confirm that HF addition did not promote microbial growth and that all formulations met the microbiological safety criteria for bakery products. The absence of contamination highlights the effectiveness of the applied baking and handling conditions, ensuring hygienic stability across all samples.

No statistical analyses (ANOVA, PCA, or AHC) were applied, as the data served descriptively to confirm product safety. The results agree with previous studies reporting that hemp flour enrichment does not compromise the microbiological stability of baked goods [23,50].

### 3.4. Physicochemical Analyses

The physicochemical composition of bakery products plays a crucial role in determining their nutritional value, texture, and overall quality. The incorporation of HF into muffins is expected to influence key parameters, including moisture content, ash content, total fiber, protein, fat, and sugar levels, thereby affecting both functional and sensory properties.

In this study, physicochemical analyses were conducted on muffins formulated with WF and WWF, with HF concentrations ranging from 5% to 20%. These analyses provide a comprehensive understanding of how HF impacts the composition of muffins, offering insights into potential nutritional improvements and formulation adjustments required for product optimization.

Moisture content is essential for product freshness and shelf stability, as it affects microbial growth and texture. Ash content represents the total mineral content, reflecting the presence of essential micronutrients. Total fiber content is a key nutritional factor, with HF contributing additional dietary fiber, which is beneficial for digestion and overall health. Protein content influences the structural and nutritional quality of the muffins, with HF being a high-protein ingredient. Fat content contributes to texture, mouthfeel, and energy value, with variations depending on the ingredient composition. Sugar content affects the perception of sweetness and caramelization reactions, which in turn impact flavor and color.

Table 6 summarizes the physicochemical analyses, emphasizing the influence of HF on both the nutritional composition and quality attributes of the muffins.

The physicochemical properties of muffins varied significantly with the addition of HF.

Moisture content exhibited a clear decreasing trend with increasing levels of hemp flour (HF). The highest value was observed in the control sample containing only wheat flour (C-WF), at 17.59%, while the lowest moisture content, 10.76%, was recorded in the sample with 20% HF substitution in the whole wheat formulation (C-WWF), representing a 38.8% reduction. This decline is primarily attributed to the high dietary fiber content of HF—particularly cellulose, hemicellulose, and lignin—which have strong water-binding capacities, thereby reducing the amount of free water available in the muffin matrix. Furthermore, the partial replacement of wheat flour with HF weakens the gluten network, diminishing its ability to retain moisture and resulting in a firmer, drier crumb texture.

Ash content, indicative of the total mineral concentration in the muffins, increased consistently with higher levels of hemp flour (HF) incorporation, rising from 0.95% in the control sample (C-WF) to 1.67% in the 20% HF sample (C-WWF), representing a 75.8% increase. This trend is attributed to the naturally high mineral content of HF, which is particularly rich in essential minerals, including calcium, magnesium, phosphorus, potassium, and iron. From a nutritional perspective, this increase is advantageous, as these minerals contribute to critical physiological functions, including bone health, enzymatic activity, and muscle function.

Crude fiber content showed the most significant increase among the parameters analyzed, rising from 2.79% in the control sample (C-WF) to 7.59% in the 20% HF formulation (C-WWF), representing a 172% improvement. This substantial enhancement highlights hemp flour’s (HF) effectiveness as a valuable source of dietary fiber. HF contains both insoluble fibers (such as cellulose and lignin) and soluble fibers (including pectins and gums), which contribute to digestive health while also influencing water absorption, gel formation, and viscosity within the food matrix. The high fiber content can also affect the texture of muffins, often increase density and reduce tenderness, which may, in turn, influence sensory acceptability.

Protein content increased steadily with the incorporation of hemp flour (HF), rising from 6.89% in the control sample (C-WF) to 9.95% in the 20% HF formulation (C-WWF), reflecting a 44.4% improvement. This trend is consistent with the naturally high protein content of HF, which is composed primarily of edestin and albumin—two highly digestible storage proteins. These proteins provide essential amino acids such as arginine, lysine, and methionine, contributing to the nutritional value of HF as a plant-based alternative to wheat flour (WF). However, unlike gluten proteins (gliadin and glutenin), hemp proteins lack strong elastic and cohesive properties, which may negatively affect the structural integrity of the muffins by limiting gas retention and reducing volume expansion during baking.

In contrast to other nutritional parameters, fat content remained relatively stable across all samples, fluctuating between 26.08% in the 5% HF formulation (C-WF) and 27.41% in the control sample, with a maximum variation of only 5.1%. This suggests that the partial replacement of wheat flour (WF) with hemp flour (HF) did not significantly alter the overall lipid content of the muffins. Although HF is known for its high concentration of polyunsaturated fatty acids (PUFAs), particularly omega-3 (α-linolenic acid) and omega-6 (linoleic acid), the relatively unchanged fat content indicates consistent lipid retention during baking. The minor variations observed are likely due to the distribution of lipids within the flour matrix and potential interactions with fiber and protein components.

Sugar content exhibited minor fluctuations across the formulations, peaking at 39.95% in the 5% HF sample (C-WF) and reaching a minimum of 27.76% in the 5% HF whole wheat formulation (C-WWF), corresponding to a 30.5% decrease at the lowest value. This variation follows a pattern of initial increase at low HF concentrations, followed by a gradual decline as HF levels rise. These changes may be attributed to interactions between dietary fiber and sugars, as fiber can influence sugar solubility, delay sugar release, and interfere with Maillard reaction dynamics during baking. Additionally, higher fiber content may lead to increased water absorption, which in turn reduces the dissolution and availability of sugars in the final product.

Overall, the incorporation of hemp flour (HF) significantly improved the nutritional profile of muffins by increasing their fiber, protein, and mineral content. These enhancements are primarily attributed to the chemical composition of HF—its high dietary fiber promotes water absorption, its proteins affect gluten functionality, and its mineral richness elevates ash content. However, these nutritional gains are accompanied by a reduction in moisture content, which can negatively influence textural and sensory properties by increasing density and reducing tenderness. Therefore, careful formulation adjustments are necessary to mitigate these effects and ensure that the final product remains acceptable in terms of both texture and palatability.

The PCA explained 92.74% of the total variance across the first two principal components (F1 = 74.29%, F2 = 18.45%). Ash, protein, and crude fiber were positively correlated with F1, indicating strong contributions to the variability between samples, while moisture and sugar were inversely related. The score plot revealed that samples with higher HF addition (especially 15–20% HF in WWF samples) clustered toward the positive side of F1, reflecting their higher nutritional values (fiber, protein, ash), whereas control samples and low HF variants of WF samples were more associated with higher moisture and sugar content.

The AHC grouped the samples into distinct clusters based on compositional similarities. Control samples formed separate branches from HF-enriched samples, indicating clear compositional differentiation. The clustering also showed that most HF-containing WWF and WF samples (especially at 10–20%) shared similar nutritional profiles.

Hemp flour (HF) enrichment had a significant effect on the physicochemical properties of muffins (*p* < 0.05). Moisture content decreased as HF levels increased, with the lowest values observed at 20% HF, while the control samples (WF and WWF) exhibited the highest moisture. Sugar content showed an opposite trend, being significantly higher in the 5% HF muffins and lowest at 15–20% HF, indicating that higher HF levels reduce the available sugars in the formulation. Protein content increased steadily with HF substitution, peaking at 20% HF, which was significantly higher compared to both WF and WWF controls. Similarly, crude fiber and ash contents increased as HF levels rose, with the 20% HF muffins showing the highest values, confirming HF’s role in boosting fiber and mineral content. Among the two base flours, WWF muffins naturally exhibited higher baseline fiber and ash, but WF muffins showed a more pronounced improvement with HF substitution. Overall, the incorporation of up to 20% HF substantially enhanced the nutritional quality of muffins without negatively impacting sugar balance or moisture retention, demonstrating the potential of HF to create nutritionally superior bakery products.

The addition of hemp flour (HF) significantly improved the nutritional composition of both WF and WWF muffins, particularly at 20% HF, which showed the highest protein, fiber, and ash contents (*p* < 0.05). These results align with those of [24], who reported similar increases in protein and dietary fiber when substituting wheat flour with 10–30% HF in bread formulations. Ref. [50] also observed proportional increases in protein and mineral content with HF enrichment, confirming the high nutrient density of hemp-based ingredients. Likewise, [15] found substantial rises in protein and fiber in gluten-free cakes enriched with HF, though higher substitution levels (>50%) negatively affected product structure and sensory quality. In our study, sugar content decreased as HF levels increased, which is consistent with the lower carbohydrate content of HF compared to wheat flour and parallels the findings of [23], who reported a similar decrease in carbohydrate levels in HF-enriched breads. These similarities across studies confirm that HF is an effective fortifying agent for enhancing the nutritional value of baked products.

HF addition improved the nutritional composition of both WF and WWF muffins by increasing protein, fiber, and ash contents. Relative increases were more pronounced in WF muffins due to their initially lower baseline levels, whereas WWF muffins reached the highest absolute nutrient values, particularly at 20% HF (Table 6). These results indicate that hemp flour contributes positively to the nutritional enrichment of both matrices, with slightly greater overall benefits in WWF-based formulations.

## 4. Conclusions

This study highlights the potential of hemp flour (HF) as a valuable ingredient for developing nutritionally enriched muffins. The gradual substitution of WF and WWF with HF at levels ranging from 5% to 40% resulted in notable improvements in the nutritional composition, particularly in dietary fiber, which increased by more than 170% at 20% HF. Protein and ash contents also rose proportionally, reflecting the high nutrient density of HF.

Physicochemical analysis showed a consistent decrease in moisture content with increasing HF levels, likely due to the strong water-binding capacity of dietary fibers such as cellulose, hemicellulose, and lignin. These changes, combined with a weakened gluten network, contributed to firmer textures and darker crumb color at higher substitution rates. WWF muffins naturally exhibited higher initial fiber and mineral levels, while WF muffins showed a more pronounced relative increase when enriched with HF, underscoring the influence of base flour composition on final product characteristics.

Microbiological evaluation confirmed that all muffin samples met food safety standards, with no significant growth of harmful microorganisms. Standard hygienic and baking practices effectively preserved microbial stability throughout production.

Sensory analysis demonstrated that flour type significantly affected consumer perception. Muffins containing up to 15% HF maintained acceptable organoleptic qualities for both flour types. However, WWF muffins enriched with 20% HF achieved the highest sensory ratings, combining enhanced nutritional benefits with good acceptability. In contrast, WF muffins at higher HF levels (≥20%) were perceived as denser and darker, with a more pronounced hemp flavor that slightly reduced consumer preference. These findings indicate that WWF provides a more compatible matrix for HF incorporation in bakery products.

In conclusion, HF offers clear advantages as a sustainable, plant-based ingredient for functional food development. It improves the protein, fiber, and mineral content of muffins, while the type of base flour plays a decisive role in determining texture, sensory quality, and overall acceptability. Future research should focus on optimizing formulations to achieve a balance between nutritional enhancement and sensory appeal, with particular attention paid to moisture retention and improving the palatability of higher HF levels in WF-based products.

## Figures and Tables

**Table 1:** Formulation of muffin samples prepared with wheat flour and whole wheat flour supplemented with different concentrations of hemp flour.

Sample	Flour %			Sugar (%)	Butter (%)	Eggs (%)	Baking Soda (%)	Flavor (%)
	Wheat	Whole Wheat	Hemp					
C–WF ^1^	30.44	-	-	22.83	22.83	22.83	0.76	0.30
5% HF ^3^	28.92	-	1.52	22.83	22.83	22.83	0.76	0.30
10% HF	27.40	-	3.04	22.83	22.83	22.83	0.76	0.30
15% HF	25.88	-	4.57	22.83	22.83	22.83	0.76	0.30
20% HF	24.35	-	6.09	22.83	22.83	22.83	0.76	0.30
30% HF	21.31	-	9.13	22.83	22.83	22.83	0.76	0.30
40% HF	18.26	-	12.18	22.83	22.83	22.83	0.76	0.30
C–WWF ^2^	-	30.44	-	22.83	22.83	22.83	0.76	0.30
5% HF	-	28.92	1.52	22.83	22.83	22.83	0.76	0.30
10% HF	-	27.40	3.04	22.83	22.83	22.83	0.76	0.30
15% HF	-	25.88	4.57	22.83	22.83	22.83	0.76	0.30
20% HF	-	24.35	6.09	22.83	22.83	22.83	0.76	0.30
30% HF	-	21.31	9.13	22.83	22.83	22.83	0.76	0.30
40% HF	-	18.26	12.18	22.83	22.83	22.83	0.76	0.30

^1^ C-WF—control sample with WF. ^2^ C-WWF—control sample with WWF. ^3^ HF—the variation in concentrations of HF between 5% and 40%.

**Table 2:** Consumer preferences rankings for muffin samples with wheat flour and whole wheat flour supplemented with different hemp flour concentrations.

Samples			Choices				Estimated Preference %
Code	WF %	HF %	First	Second	Third	Fourth	
426	95	5	9	7	7	5	82.35
951	90	10	7	10	3	5	73.53
159	85	15	10	5	5	7	79.41
684	80	20	3	4	11	9	79.41
357	70	30	2	2	5	4	38.24
753	60	40	3	6	3	4	47.06
**Code**	**WWF %**	**HF %**	**First**	**Second**	**Third**	**Fourth**	
351	95	5	12	10	5	1	82.35
934	90	10	11	5	6	3	73.53
671	85	15	3	8	12	5	82.35
248	80	20	1	5	9	12	79.41
349	70	30	2	4	2	9	50.00
761	60	40	5	2	0	4	32.35

Note: Estimated preferences (%) = (Total Selections ÷ Total participants) × 100.

**Table 3:** Descriptive sensory scores for muffin samples prepared with wheat flour and whole wheat flour supplemented with different hemp flour concentrations (related to Figure 4).

Samples	Attribute						
	Exterior Aspect	Interior Aspect	Core Texture	Smell	First Bite	Taste and Aroma	After-Taste
C–WF ^1^	1.6 ± 0.6 ^b^	2.3 ± 1.2 ^b^	3.5 ± 1.2 ^b^	2.6 ± 1.5 ^b^	2.8 ± 1.4 ^b^	2.5 ± 1.4 ^b^	3.1 ± 1.3 ^b^
5% HF ^3^	2.5 ± 0.6 ^b^	2.4 ± 1.1 ^b^	3.1 ± 1.0 ^b^	2.7 ± 1.3 ^b^	2.8 ± 1.2 ^b^	2.4 ± 1.3 ^b^	2.9 ± 1.1 ^b^
10% HF	3.3 ± 0.7 ^a,b^	2.9 ± 1.2 ^a,b^	4.0 ± 1.0 ^a,b^	3.1 ± 1.2 ^a,b^	3.2 ± 1.2 ^a,b^	2.5 ± 1.2 ^b^	3.0 ± 1.1 ^b^
15% HF	3.6 ± 0.7 ^a,b^	3.0 ± 1.1 ^a,b^	3.7 ± 1.0 ^a,b^	3.0 ± 1.2 ^a,b^	3.0 ± 1.1 ^a,b^	2.4 ± 1.2 ^b^	3.1 ± 1.1 ^b^
20% HF	3.9 ± 0.8 ^a^	3.0 ± 1.2 ^a,b^	3.4 ± 1.0 ^a,b^	3.2 ± 1.3 ^a^	2.9 ± 1.3 ^a,b^	2.4 ± 1.3 ^b^	3.1 ± 1.3 ^b^
C–WWF ^2^	1.9 ± 0.7 ^b^	2.5 ± 1.3 ^b^	3.7 ± 0.8 ^b^	2.5 ± 1.3 ^b^	3.1 ± 1.2 ^b^	2.4 ± 1.2 ^b^	2.3 ± 1.0 ^b^
5% HF	3.0 ± 0.7 ^a,b^	2.5 ± 1.1 ^b^	2.0 ± 1.0 ^b^	2.9 ± 1.4 ^b^	2.8 ± 1.3 ^b^	2.7 ± 1.5 ^b^	3.3 ± 1.4 ^a,b^
10% HF	3.2 ± 0.6 ^a,b^	3.0 ± 1.0 ^a,b^	3.2 ± 1.0 ^a,b^	3.0 ± 1.2 ^a,b^	3.0 ± 1.1 ^a,b^	2.5 ± 1.2 ^b^	2.5 ± 1.0 ^b^
15% HF	3.8 ± 0.7 ^a,b^	3.1 ± 1.0 ^a,b^	3.4 ± 0.9 ^a,b^	3.2 ± 1.4 ^a,b^	3.0 ± 1.2 ^a,b^	2.5 ± 1.2 ^b^	2.9 ± 0.9 ^a,b^
20% HF	4.6 ± 0.6 ^a^	3.3 ± 1.3 ^a,b^	3.7 ± 1.1 ^a^	3.6 ± 1.2 ^a^	3.2 ± 1.3 ^a,b^	2.7 ± 1.4 ^b^	3.3 ± 1.3 ^a^

^1^ C-WF—control sample with WF. ^2^ C-WWF—control sample with WWF ^3^ HF—the variation in concentrations of HF between 5% and 20%. Different letters in the same row indicate statistically significant differences between samples (Tukey test, *p* < 0.05).

**Table 4:** Mean values and standard deviations (SD) of key texture parameters of muffin samples prepared with wheat flour and whole wheat flour with different hemp seed flour concentrations.

Sample	Firmness (N)	Cohesiveness	Elasticity	Gumminess
C–WF ^1^	6.29 ± 1.47	0.2 ± 0.13	1.62 ± 0.38	1.84 ± 1.15
5% HF ^3^	4.08 ± 0.19	0.12 ± 0.08	1.56 ± 0.14	0.74 ± 0.50
10% HF	7.81 ± 1.36	0.18 ± 0.18	1.55 ± 0.93	1.72 ± 1.82
15% HF	5.06 ± 0.37	0.06 ± 0.07	1.20 ± 0.38	0.50 ± 0.63
20% HF	5.04 ± 0.06	−0.01 ± 0.22	2.42 ± 0.30	−1.53 ± 2.93
C–WWF ^2^	5.28 ± 1.95	−0.67	3.52	−8.67
5% HF	4.10 ± 0.50	0.22 ± 0.03	1.63 ± 0.024	1.44 ± 0.24
10% HF	7.89 ± 1.28	0.14 ± 0.05	1.95 ± 0.12	2.19 ± 0.96
15% HF	6.87 ± 0.56	0.15 ± 0.02	2.04 ± 0.32	2.12 ± 0.14
20% HF	8.05 ± 2.70	0.13 ± 0.02	1.94 ± 0.93	2.36 ± 1.37

^1^ C-WF—control sample with WF. ^2^ C-WWF—control sample with WWF. ^3^ HF—the variation in concentrations of HF between 5% and 20%.

**Table 5:** Yeast, Mold, and *Enterobacteriaceae* counts (CFU/g) in muffin samples prepared with wheat flour and whole wheat flour supplemented with different concentrations of hemp flour.

Sample	Yeast and Molds (CFU/g)	*Enterobacteriaceae* (CFU/g)
C–WF ^1^	<10	<10
5% HF ^3^	<10	<10
10% HF	<10	<10
15% HF	<10	<10
20% HF	<10	<10
C–WWF ^2^	<10	<10
5% HF	<10	<10
10% HF	<10	<10
15% HF	<10	<10
20% HF	<10	<10

^1^ C-WF—control sample with WF. ^2^ C-WWF—control sample with WWF. ^3^ HF—the variation in concentrations of HF between 5% and 20%.

**Table 6:** Physicochemical properties of muffin samples prepared with wheat flour and whole wheat flour supplemented with different concentrations of hemp seed flour.

Sample	Moisture %	Ash %	Crude Fiber %	Protein %	Fat %	Sugar %
C–WF ^1^	17.59 ± 0.014 ^a^	0.95 ± 0.028 ^c^	2.79 ± 0.099 ^e^	6.89 ± 0.028 ^c^	27.41 ± 0.014	32.05 ± 0.007 ^b^
5% HF ^3^	14.58 ± 0.014 ^b^	1.10 ± 0.064 ^c^	3.98 ± 0.191 ^d^	8.07 ± 0.085 ^d^	26.08 ± 0.219	39.95 ^b^
10% HF	14.10 ± 0.014 ^b^	1.25 ± 0.021 ^b^	5.45 ± 0.233 ^c^	8.59 ± 0.021 ^b^	26.24 ± 0.191	31.40 ± 0.014 ^b^
15% HF	14.62 ^b^	1.35 ± 0.042 ^b^	4.64 ± 0.269 ^c^	8.68 ± 0.078 ^b^	26.18 ± 0.014	29.07 ± 0.007 ^c^
20% HF	14.45 ± 0.007 ^c^	1.44 ± 0.071 ^a^	5.87 ± 0.099 ^b^	9.26 ± 0.049 ^b^	26.09 ± 0.049	29.07 ^c^
C–WWF ^2^	12.88 ± 0.007 ^a^	1.35 ± 0.014 ^b^	3.64 ± 0.304 ^d^	8.82 ± 0.007 ^b^	26.66 ± 0.042	31.07 ± 0.007 ^b^
5% HF	13.80 ^b^	1.38 ± 0.014 ^b^	4.38 ± 0.019 ^c^	9.29 ± 0.042 ^a^	26.28 ± 0.014	27.76 ^c^
10% HF	12.78 ± 0.007 ^b^	1.42 ± 0.035 ^b^	5.66 ± 0.453 ^c^	9.54 ^a^	26.62 ± 0.071	30.40 ± 0.007 ^b^
15% HF	13.10 ± 0.014 ^b^	1.59 ± 0.014 ^a^	7.00 ± 0.247 ^a^	9.81 ± 0.021 ^a^	26.55 ± 0.028	32.33 ± 0.071 ^a^
20% HF	10.76 ± 0.007 ^c^	1.67 ± 0.007 ^a^	7.59 ± 0.057 ^a^	9.95 ± 0.028 ^a^	27.02 ± 0.049	30.73 ± 0.007 ^b^

^1^ C-WF—control sample with WF. ^2^ C-WWF—control sample with WWF. ^3^ HF—the variation in concentrations of HF between 5% and 20%. Different letters within the same column indicate significant differences among samples according to Tukey’s HSD test (*p* < 0.05). Values with the same letter are not significantly different. Fat content showed no significant differences (*p* > 0.05), so no superscript letters are shown in that column.

## Data Availability

All data supporting the findings of this study are included in the article.

## Supplementary Materials

The following supporting information can be downloaded at: https://www.mdpi.com/article/10.3390/foods14203578/s1, Figures S1–S16: PCA and AHC analyses of sensory, textural, color, and physicochemical datasets of muffins enriched with different percentages of hemp flour.
