# Supplementary material for: Impact of Hemp Flour on the Nutritional, Sensory and Functional Characteristics of Wheat and Whole Wheat Muffins
Source: Foods. 2025 Oct 21;14(20):3578. doi: 10.3390/foods14203578 (PMC12562866; doi:10.3390/foods14203578)
Supplement: Supplementary file 1 [file foods-14-03578-s001.zip › foods-3921040-supplementary.pdf]

Supplementary Materials for:

Impact of Hemp Flour on the Nutritional, Sensory and Functional Characteristics of Wheat and Whole Wheat Muffins

Authors: Andreea – Lavinia MOCANU, Alina Alexandra DOBRE, Corina – Alexandra STROE, Cătălina – Beatrice POTERAȘ, Elena – Loredana UNGUREANU, Gabriel MUSTĂȚEA, Gabriela CRIVEANU-STAMATIE, Șerban Eugen CUCU, Sabina – Andreea BOBEA, Cristian FLOREA, Bogdan Mihai NICOLCIOIU and Raluca STAN

Correspondence: Raluca STAN

Introduction

The following supplementary figures show the PCA and AHC analyses performed on data sets obtained.

Figures

Figure S1. PCA biplot showing the relationships among sensory attributes of muffins enriched with different percentages of hemp flour. The first two principal components (F1 and F2) explain 81.46% of the total variance (F1: 51.64%, F2: 29.82%). Variables such as core texture, first bite, interior aspect, exterior aspect, and taste/aroma are plotted as active variables.

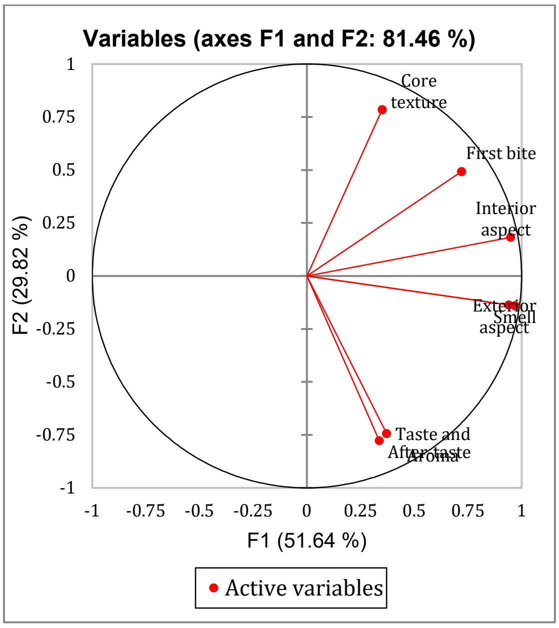

Figure S2. PCA score plot showing the distribution of muffin samples prepared with different percentages of hemp flour and control samples. C-WF: control sample with wheat flour; C-WWF: control sample with whole wheat flour; W-HF (5%, 10%, 15%, 20%): samples with wheat flour and hemp flour in different percentages; WW-HF (5%, 10%, 15%, 20%): samples with whole wheat flour and hemp flour in different percentages.

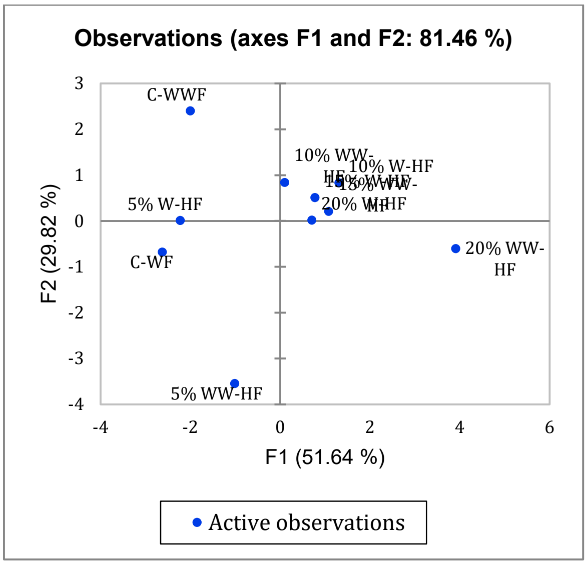

Figure S3. PCA biplot combining sensory attributes (active variables) and muffin samples (active observations) enriched with varying percentages of hemp flour. C-WF: control sample with wheat flour; C-WWF: control sample with whole wheat flour; W-HF (5%, 10%, 15%, 20%): samples with wheat flour and hemp flour in different percentages; WW-HF (5%, 10%, 15%, 20%): samples with whole wheat flour and hemp flour in different percentages.

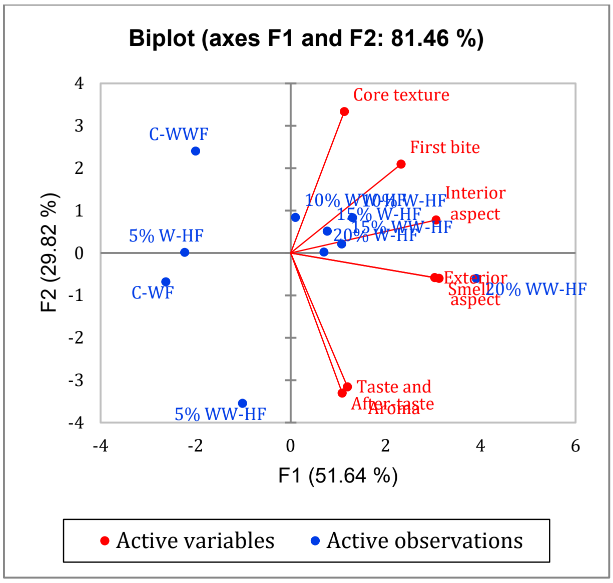

Figure S4. Agglomerative Hierarchical Clustering (AHC) dendrogram of muffin samples formulated with different percentages of hemp flour. The clustering was performed using the software with Euclidian distance as the similarity measure of Ward's method for linkage. C-WF: control sample with wheat flour; C-WWF: control sample with whole wheat flour; W-HF (5%, 10%, 15%, 20%): samples with wheat flour and hemp flour in different percentages; WW-HF (5%, 10%, 15%, 20%): samples with whole wheat flour and hemp flour in different percentages.

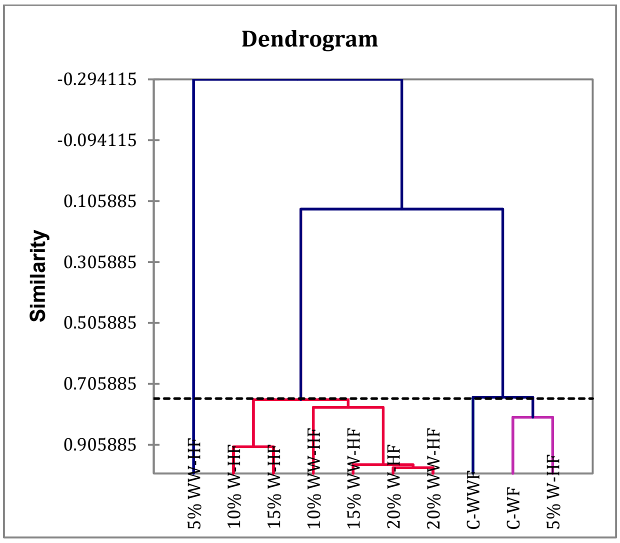

Figure S5. PCA biplot of textural parameters of muffins enriched with different percentages of hemp flour. The first two principal components (F1 and F2) explained 80.82% of the total variance (F1: 53.06%, F2: 27.76%). Textural attributes include firmness, gumminess, cohesiveness, and elasticity, presented as active variables.

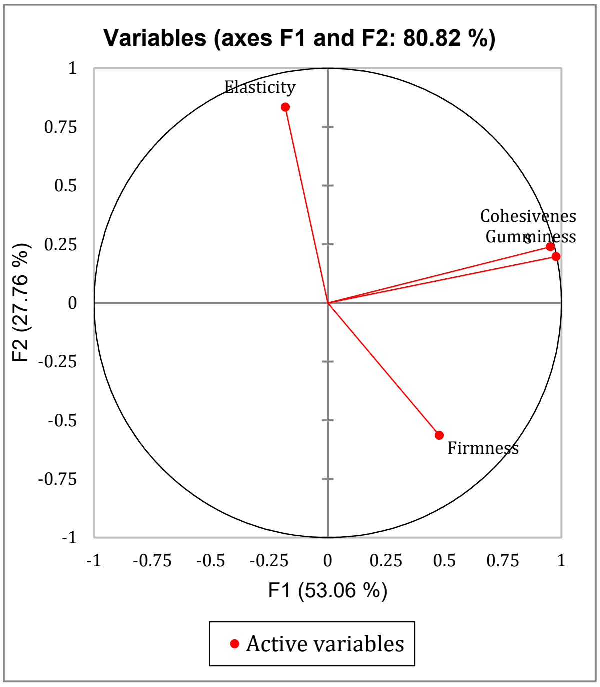

Figure S6. PCA score plot showing the distribution of muffin samples prepared with different percentages of hemp flour and control samples. C-WF: control sample with wheat flour; C-WWF: control sample with whole wheat flour; W-HF (5%, 10%, 15%, 20%): samples with wheat flour and hemp flour in different percentages; WW-HF (5%, 10%, 15%, 20%): samples with whole wheat flour and hemp flour in different percentages.

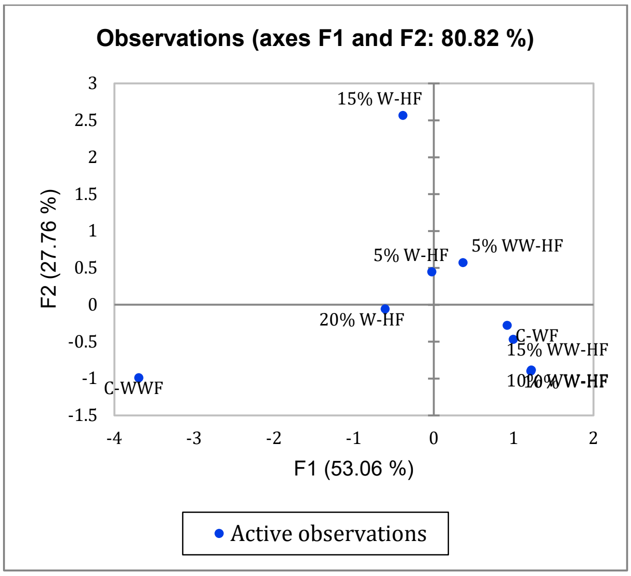

Figure S7. PCA biplot combining textural parameters (active variables) and muffin samples (active observations) enriched with varying percentages of hemp flour. C-WF: control sample with wheat flour; C-WWF: control sample with whole wheat flour; W-HF (5%, 10%, 15%, 20%): samples with wheat flour and hemp flour in different percentages; WW-HF (5%, 10%, 15%, 20%): samples with whole wheat flour and hemp flour in different percentages.

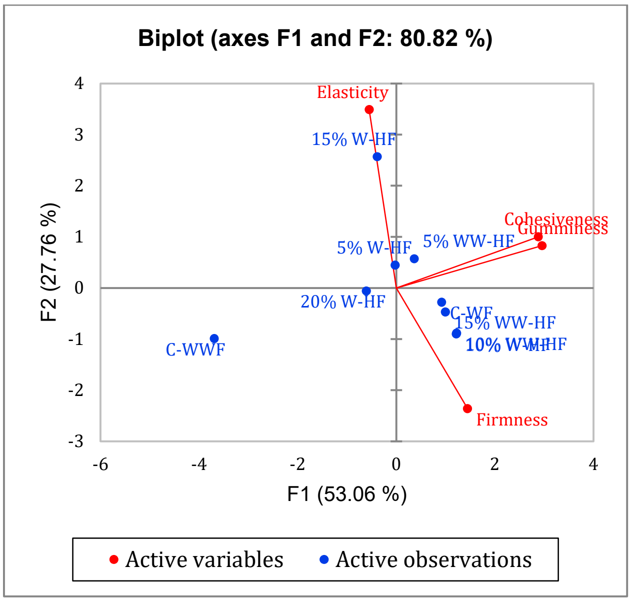

Figure S8. Agglomerative Hierarchical Clustering (AHC) dendrogram of muffin samples formulated with different percentages of hemp flour. The clustering was performed using the software with Euclidian distance as the similarity measure of Ward's method for linkage. C-WF: control sample with wheat flour; C-WWF: control sample with whole wheat flour; W-HF (5%, 10%, 15%, 20%): samples with wheat flour and hemp flour in different percentages; WW-HF (5%, 10%, 15%, 20%): samples with whole wheat flour and hemp flour in different percentages.

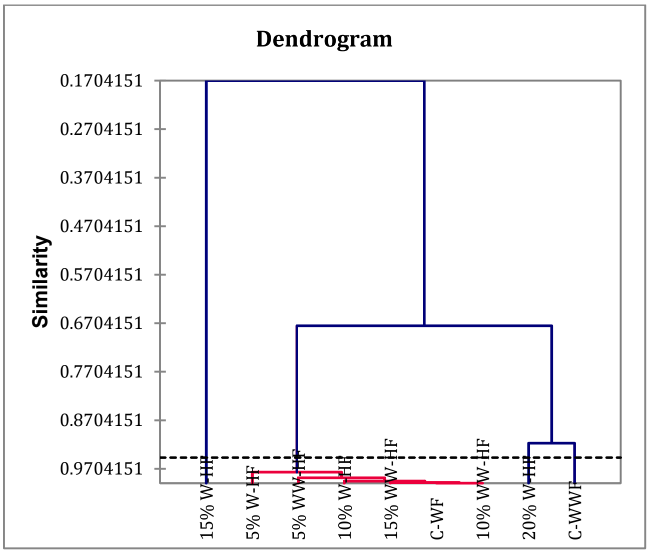

Figure S9. PCA biplot of color parameters of muffins enriched with different percentages of hemp flour. The first two principal components (F1 and F2) explained 96.05% of the total variance (F1: 62.58%, F2: 33.47%). Color attributes L*, a*, and b*, presented as active variables.

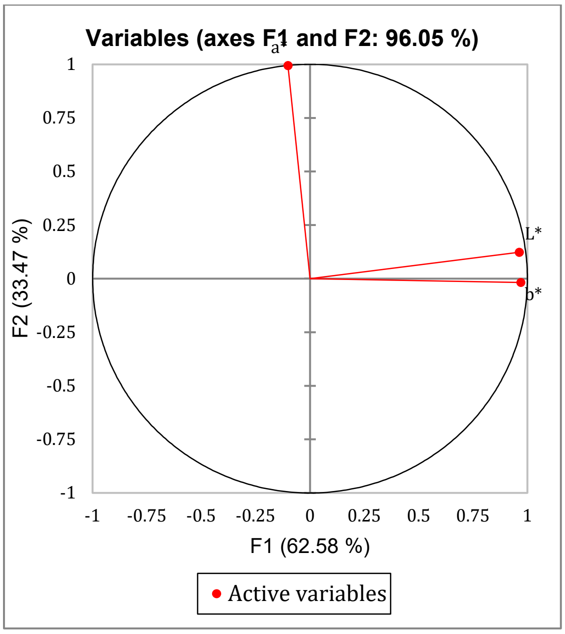

Figure S10. PCA score plot showing the distribution of muffin samples prepared with different percentages of hemp flour and control samples. C-WF: control sample with wheat flour; C-WWF: control sample with whole wheat flour; W-HF (5%, 10%, 15%, 20%): samples with wheat flour and hemp flour in different percentages; WW-HF (5%, 10%, 15%, 20%): samples with whole wheat flour and hemp flour in different percentages.

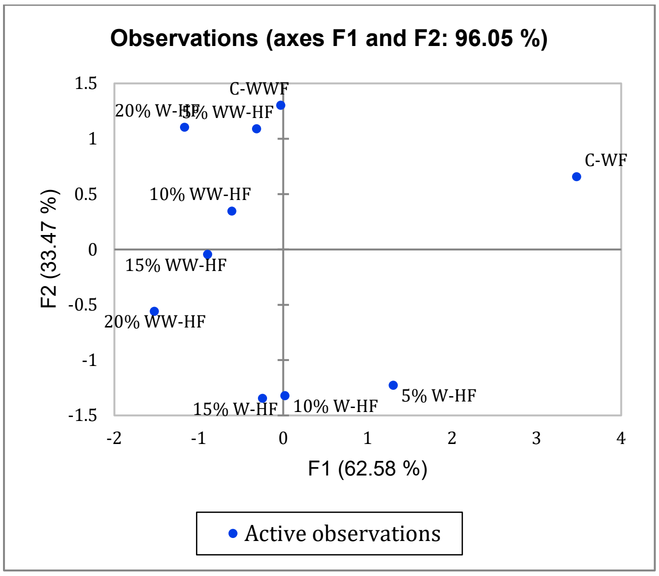

Figure S11. PCA biplot combining color parameters (active variables) and muffin samples (active observations) enriched with varying percentages of hemp flour. C-WF: control sample with wheat flour; C-WWF: control sample with whole wheat flour; W-HF (5%, 10%, 15%, 20%): samples with wheat flour and hemp flour in different percentages; WW-HF (5%, 10%, 15%, 20%): samples with whole wheat flour and hemp flour in different percentages.

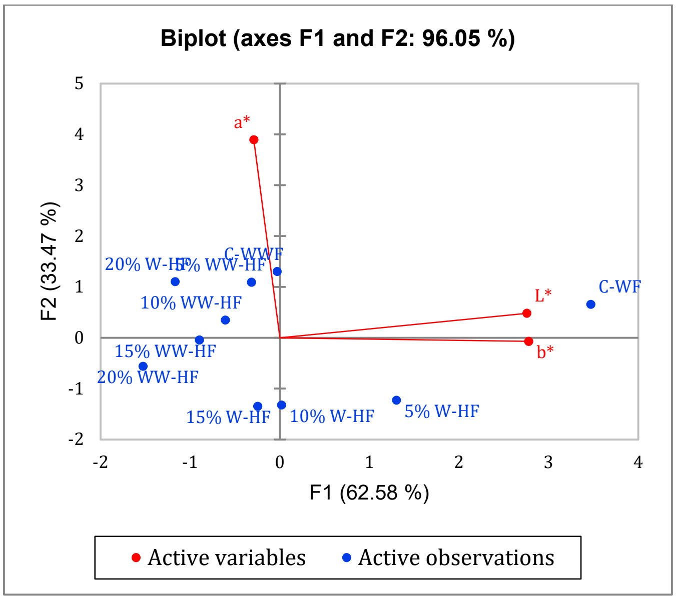

Figure S12. Agglomerative Hierarchical Clustering (AHC) dendrogram of muffin samples formulated with different percentages of hemp flour. The clustering was performed using the software with Euclidian distance as the similarity measure of Ward's method for linkage. C-WF: control sample with wheat flour; C-WWF: control sample with whole wheat flour; W-HF (5%, 10%, 15%, 20%): samples with wheat flour and hemp flour in different percentages; WW-HF (5%, 10%, 15%, 20%): samples with whole wheat flour and hemp flour in different percentages.

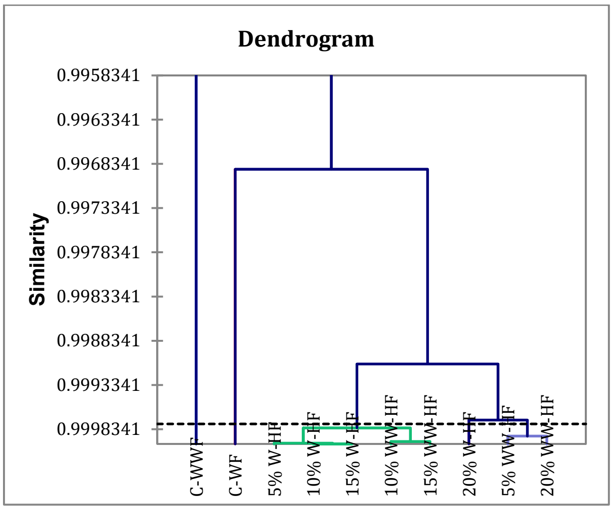

Figure S13. PCA biplot of physicochemical parameters of muffins enriched with different percentages of hemp flour. The first two principal components (F1 and F2) explained 92.74% of the total variance (F1: 74.29%, F2: 18.45%). Physicochemical attributes moisture, protein, ash, crude fiber, and sugar, presented as active variables.

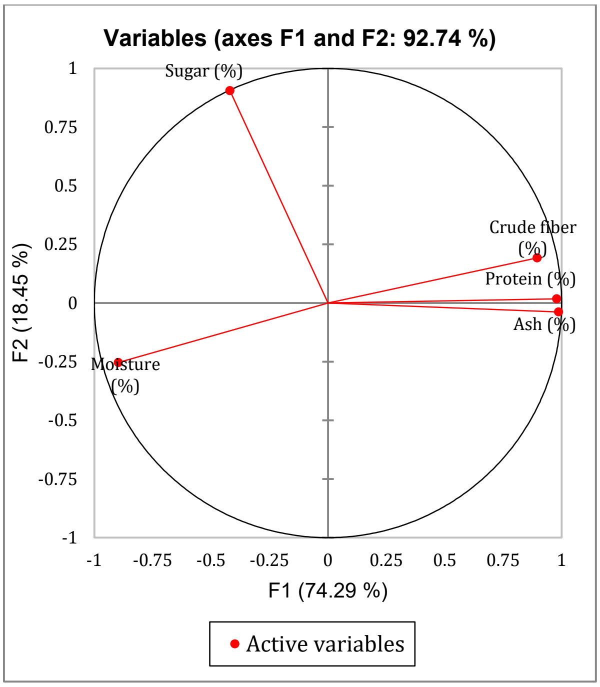

Figure S14. PCA score plot showing the distribution of muffin samples prepared with different percentages of hemp flour and control samples. C-WF: control sample with wheat flour; C-WWF: control sample with whole wheat flour; W-HF (5%, 10%, 15%, 20%): samples with wheat flour and hemp flour in different percentages; WW-HF (5%, 10%, 15%, 20%): samples with whole wheat flour and hemp flour in different percentages.

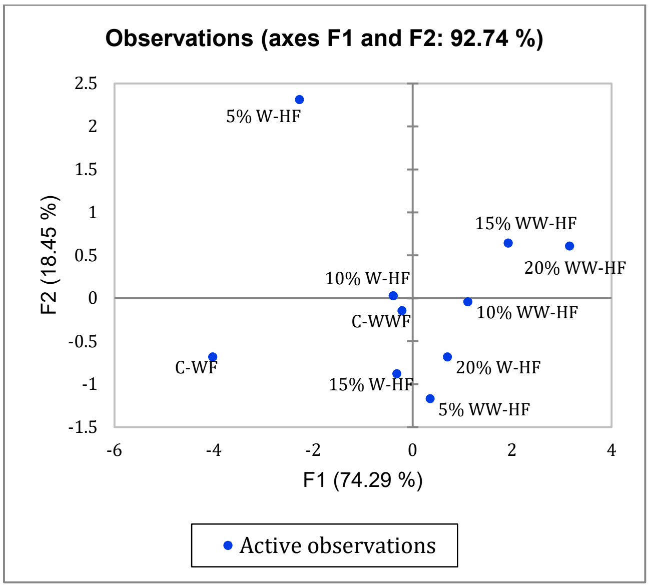

Figure S15. PCA biplot combining physicochemical parameters (active variables) and muffin samples (active observations) enriched with varying percentages of hemp flour. C-WF: control sample with wheat flour; C-WWF: control sample with whole wheat flour; W-HF (5%, 10%, 15%, 20%): samples with wheat flour and hemp flour in different percentages; WW-HF (5%, 10%, 15%, 20%): samples with whole wheat flour and hemp flour in different percentages.

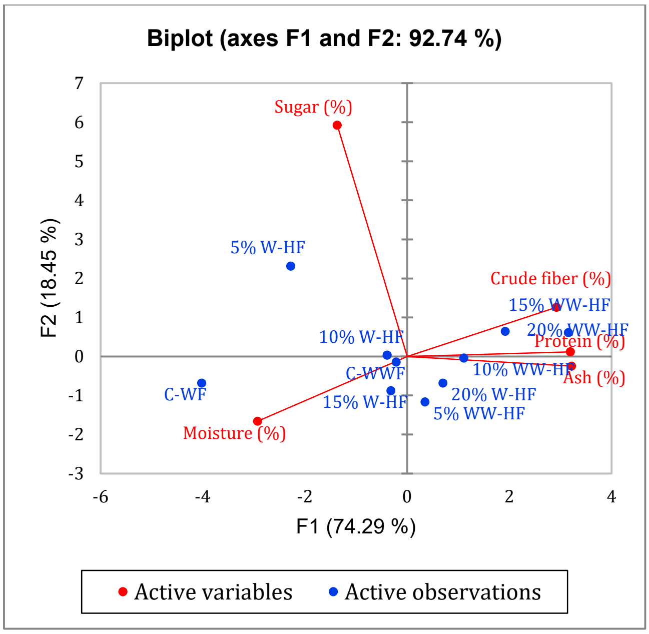

Figure S16. Agglomerative Hierarchical Clustering (AHC) dendrograms of muffin samples formulated with different percentages of hemp flour. (A) Overall clustering of samples prepared with different hemp flour concentrations. (B) Detailed view of the 5 clusters identified in panel A, showing a closer examination of relationships between sample groups. The clustering was performed using the software with Euclidian distance as the similarity measure of Ward's method for linkage. C-WF: control sample with wheat flour; C-WWF: control sample with whole wheat flour; W-HF (5%, 10%, 15%, 20%): samples with wheat flour and hemp flour in different percentages; WW-HF (5%, 10%, 15%, 20%): samples with whole wheat flour and hemp flour in different percentages.

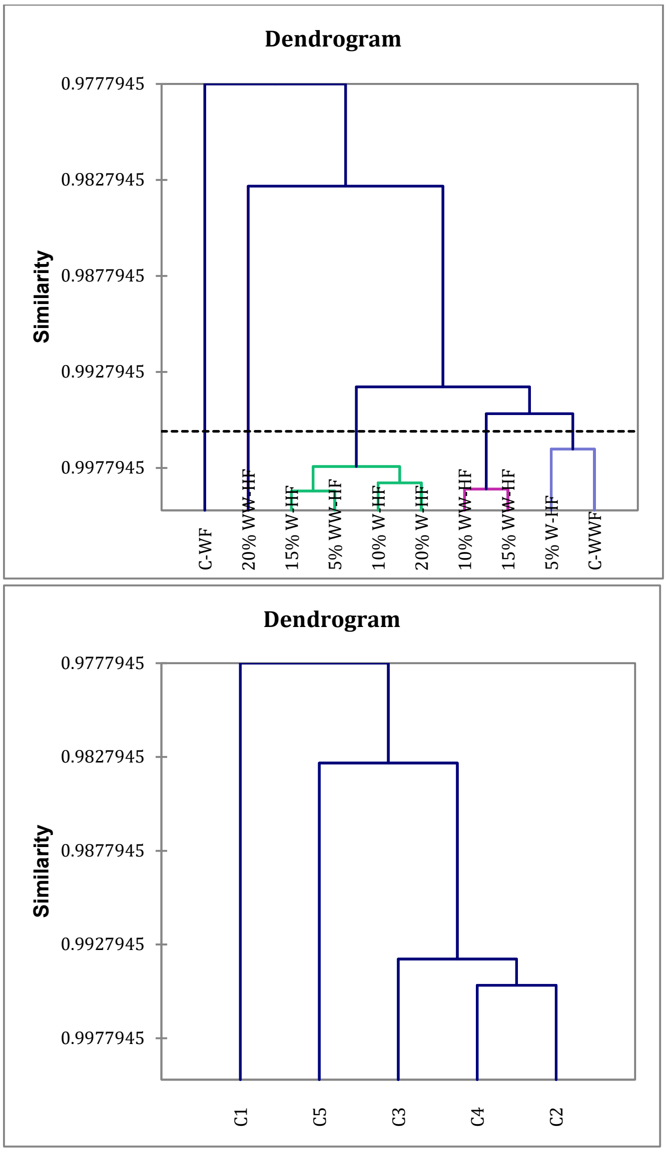

References

XLSTAT statistical software, version 2024. Addinsoft, Paris, France. Available online: http://www.xlstat.com
